# Inhibition of inflammation and oxidative stress by an imidazopyridine derivative X22 prevents heart injury from obesity

**DOI:** 10.1111/jcmm.12832

**Published:** 2016-03-28

**Authors:** Yuanyuan Qian, Yali Zhang, Peng Zhong, Kesong Peng, Zheng Xu, Xuemei Chen, Kongqin Lu, Gaozhi Chen, Xiaokun Li, Guang Liang

**Affiliations:** ^1^ Chemical Biology Research CenterSchool of Pharmaceutical SciencesWenzhou Medical UniversityWenzhouZhejiangChina; ^2^ Department of CardiologyThe 5th Affiliated Hospital of Wenzhou Medical UniversityLishuiZhejiangChina

**Keywords:** obesity‐related cardiomyopathy, imidazopyridine derivative, oxidative stress, inflammation

## Abstract

Inflammation and oxidative stress plays an important role in the development of obesity‐related complications and cardiovascular disease. Benzimidazole and imidazopyridine compounds are a class of compounds with a variety of activities, including anti‐inflammatory, antioxidant and anti‐cancer. X22 is an imidazopyridine derivative we synthesized and evaluated previously for anti‐inflammatory activity in lipopolysaccharide‐stimulated macrophages. However, its ability to alleviate obesity‐induced heart injury *via* its anti‐inflammatory actions was unclear. This study was designed to evaluate the cardioprotective effects of X22 using cell culture studies and a high‐fat diet rat model. We observed that palmitic acid treatment in cardiac‐derived H9c2 cells induced a significant increase in reactive oxygen species, inflammation, apoptosis, fibrosis and hypertrophy. All of these changes were inhibited by treatment with X22. Furthermore, oral administration of X22 suppressed high‐fat diet‐induced oxidative stress, inflammation, apoptosis, hypertrophy and fibrosis in rat heart tissues and decreased serum lipid concentration. We also found that the anti‐inflammatory and anti‐oxidative actions of X22 were associated with Nrf2 activation and nuclear factor‐kappaB (NF‐κB) inhibition, respectively, both *in vitro* and *in vivo*. The results of this study indicate that X22 may be a promising cardioprotective agent and that Nrf2 and NF‐κB may be important therapeutic targets for obesity‐related complications.

## Introduction

In the past few decades, the prevalence of obesity has increased dramatically worldwide to become a global epidemic. Obesity has been linked to a dramatic escalation of nephropathy and cardiomyopathy and is also associated with elevated risks of cardiovascular and cerebrovascular disease, hypertension, sleep disorders and dyslipidemia 1, 2. Furthermore, increasing evidence shows that obesity is associated with structural and functional changes in the heart in both humans and animal models 3, 4, 5. In the earliest postmortem investigations, subsequent autopsy findings confirmed increased heart weight and left ventricular and right ventricular hypertrophy in proportion to the degree of obesity 6, 7, 8. Myocardial changes associated with obesity are becoming increasingly recognized as obesity cardiomyopathy, a condition independent of diabetes, hypertension, coronary artery disease or other etiologies. The most important mechanisms in the development of obesity cardiomyopathy include metabolic disturbances, activation of the renin–angiotensin–aldosterone and sympathetic nervous systems, myocardial remodelling and small‐vessel disease 6.

Obesity has also become increasingly characterized as an inflammatory state, as chronic low‐grade inflammation and oxidative stress play important roles in the pathogenesis of obesity‐related complications 9, 10, 11, 12, 13. It is well known that circulating free fatty acids (FFAs) associated with obesity, including palmitic acid (PA), can cause chronic inflammation, insulin resistance and cardiovascular disease. Free fatty acids can also increase the expression of pro‐inflammatory cytokines and induce cellular oxidative stress, and it has been demonstrated that both *in vitro* and *in vivo* that FFAs can activate the nuclear factor‐kappaB (NF‐κB) pathway, subsequently increasing the expression of several pro‐inflammatory cytokines such as tumour necrosis factor (TNF)‐α, interleukin (IL)‐6 and IL‐1β 1, 2. In both human and animal models, this low‐grade inflammation, combined with oxidative stress in various organs like the heart, can manifest itself as hypertrophy, apoptosis and fibrosis 8, 14, 15.

Currently, treatment options for obesity are limited primarily to diet, exercise and lifestyle modifications, all of which have high failure rates. Few obesity drugs exist, and those that do are not very effective 16. However, as more studies confirm the role of inflammation and oxidative stress in the development and progression of obesity‐related complications, molecules with anti‐inflammatory and antioxidant properties may increase the efficacy of current treatment protocols for obesity‐, FFA‐, and high‐fat diet (HFD)‐induced injury.

**Figure 1:**
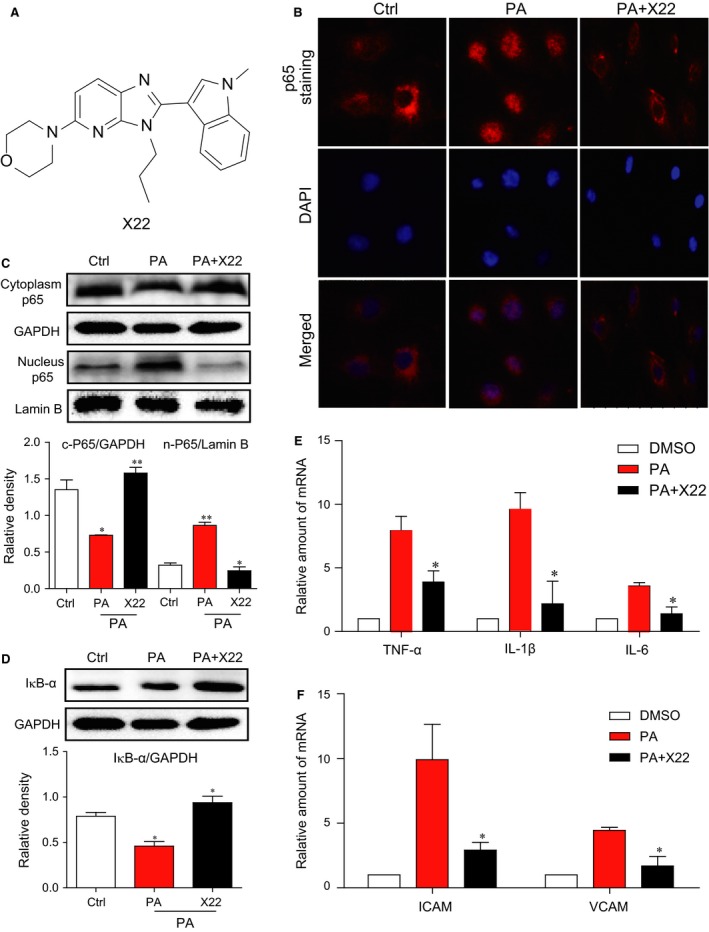
X22 mitigated NF‐κB‐mediated inflammatory response in PA‐induced H9c2 cells. (**A**) The chemical structure of X22. (**B**–**D**) H9c2 cells were pretreated with X22 (20 μM) for 1 hr and then incubated with PA (500 μM) for 45 min. (**B**) p65 immunofluorescence staining. (**C** and **D**) The Western blot analysis detected the nuclear and cytoplasmic protein levels of p65 (**C**) and the protein levels of IκB‐α (**D**). (**E** and **F**) H9c2 cells were pretreated with X22 (20 μM) for 1 hr and then incubated with PA (500 μM) for 6 hrs. The mRNA expression of inflammatory cytokines and adhesion molecules were detected by real‐time qPCR assay (*n* = 4 for each experiment; *P* < 0.05, **P < 0.01).

Previous studies have shown that imidazopyridines possess anti‐inflammatory properties. Ashwell *et al*. reported the discovery and optimization of a series of imidazopyridines to be effective inhibitors of protein kinase B, which functions as a key signalling node in cell proliferation, survival and inflammatory stress response 17, 18, 19. Other studies have also described imidazopyridines as potential antioxidant and anti‐cancer agents 20, 21. Building off of this previous evidence, our group synthesized a series of new imidazopyridine derivatives and screened them for anti‐inflammatory activities. Of the 23 imidazopyridine derivatives tested, X22 was among the few that showed significant potential, inhibiting lipopolysaccharide (LPS)‐induced TNF‐α and IL‐6 production in macrophages 17, and because of these results, X22 was targeted for further analysis (Fig. 1A). While preliminary studies done by our group had found that X22 can inhibit LPS‐induced inflammatory response in macrophages, whether or not it can protect against FFA‐induced inflammation and oxidative stress was unclear. Therefore, to validate our ideas, we explored the effects of X22 *in vitro* in rat heart H9c2 cells. Furthermore, we explored whether or not X22′s *in vitro* results can translate to *in vivo* using a HFD rat obesity model to investigate if X22 can inhibit FFA‐induced myocardial injury including cardiomyocyte hypertrophy, fibrosis and apoptosis.

## Materials and methods

### Chemicals and reagents

Palmitate (PA) was purchased from Sigma‐Aldrich (St. Louis, MO, USA). Stock solutions of 5 mM PA/10% bovine serum albumin (BSA) were prepared and stored at 4°C. Stock solutions were heated for 15 min. at 55°C and then cooled to room temperature prior to use. The dilution of PA/BAS solution to 500 μM of PA concentration was used in cellular experiments. X22 was dissolved in dimethyl sulfoxide (DMSO) for *in vitro* experiments and in carboxymethylcellulose sodium (CMCNa; 0.5%) for *in vivo* experiments. Antibodies used in the experiments were purchased from the following suppliers: Nrf2, Bcl‐2‐like protein 4 (Bax), B‐cell lymphoma 2 (Bcl2), NF‐κB p65, inhibitor of κB (IκB), CD68, cleaved‐poly (ADP‐ribose) polymerase (PARP) and A‐type natriuretic peptide (ANP), transforming growth factor (TGF)‐β, Collagen IV from Santa Cruz Biotechnology (Santa Cruz, CA, USA), TNF‐α from Abcam (Cambride, MA, USA), anti‐cleavaged caspase‐3 and 3‐NT from Cell Signaling Technology (Danvers, MA, USA) and horseradish peroxidase‐conjugated anti‐rabbit secondary antibodies from Santa Cruz. Enhanced chemiluminescence (ECL) reagent and fluorescein isothiocyanate (FITC) annexin V apoptosis detection kit were obtained from Beyotime (Beijing, China).

### Cell culture and treatment

All cellular studies were conducted with H9c2 rat heart‐derived embryonic myocytes (CRL‐1446; American Type Culture Collection, Manassas, VA, USA) cultured using DMEM/F12 supplemented with 10% (v/v) foetal bovine serum, 100 U/ml penicillin G, 100 mg/ml streptomycin and 2 mM L‐glutamine. Cells were incubated at 37°C with 5% CO_2_ and 95% air. For all experiments, cells were plated in six‐well plates or 35‐mm culture dishes at 5.0 × 10^4^ cells/cm^2^. For cell stimulation, X22 was added 1 hr prior to PA after the H9c2 cells adherence for 12 hrs. Then, the old medium was removed and replaced with new medium. The cells were stimulated with 500 μm PA. Cells were incubated for the indicated time points and then harvested for biochemical or molecular assays. All experiments were repeated at least three times to demonstrate their reproducibility.

### Determination of intracellular ROS

Dihydroethidium (DHE) and 2,7‐dichlorodihydrofluorescein diacetate (DCFH‐DA) assay: The presence of free radicals in the H9c2 cells after PA stimulation was determined using DHE or DCFH‐DA assay kits, respectively (Beyotime, Nanjing, China). After the H9c2 cells adherence for 12 hrs, X22 was added 1 hr prior to PA in all experiments. Immediately after 500 μM PA stimulation, cells were washed with PBS, incubated in fresh culture medium containing 2 μM DHE or 2 μM DCFH‐DA for 30 min. at 37°C and washed three times. Fluorescence intensity was measured with a fluorescence microscope, with excitation wavelengths of 535 nm or 488 nm. We then collected cells for flow cytometry using BD FACSCalibur^™^ (BD Biosciences, San Jose, CA, USA) and Cell Quest software.

#### GSH/GSSG assay

The cells were stimulated with 0.5 mg/ml LPS for 8 hrs or 500 μm PA for 10 hrs. After treatment, cells were lysed and the 30 μl of collected proteins were performed to reduced glutathione (GSH)/oxidized glutathione (GSSG) determination using a commercial GSH/GSSG assay kit (Beyotime Biotech, Nantong, China) according to manufacturer's instruction.

### Immunofluorescence assay for NF‐κB p65 and TGF‐β

Immediately after stimulation, cells were fixed with 4% paraformaldehyde and permeabilized with 100% methanol at −20°C for 5 min. After fixation and permeabilization, cells were washed twice with PBS containing 1% BSA and then incubated with primary antibodies for transcription factor p65 or TGF‐β (Santa Cruz Biotechnology) overnight at 4°C, followed by FITC‐ or phycoerythrin (PE)‐conjugated secondary antibody (Santa Cruz Biotechnology). Then, the cells were counterstained with 4′,6‐diamidino‐2‐phenylindole (DAPI). The stained cells were viewed under fluorescence microscope (200× amplification; Nikon, Tokyo, Japan).

### Preparation of nuclear extracts

Nuclear protein extraction from H9c2 cells was done by using nuclear protein extraction kit (Beyotime Biotech) according to the manufacturer's instructions. The protein concentration was determined using Bio‐Rad protein assay reagent (Hercules, CA, USA). The nuclear extract (15 μg protein) was used for the Western immunoblot analysis.

### Morphological analysis and rhodamine‐phalloidin staining

Immediately after stimulation, H9c2 cells were fixed with 4% paraformaldehyde followed by taking phage micrograph using a light microscope (400× amplification; Nikon). Then, the cells were permeabilized with 0.1% Triton‐X100 and stained with rhodamine‐phalloidin at a concentration of 50 μg/ml for 30 min. at room temperature and then washed with PBS and visualized by fluorescence microscope (400× amplification; Nikon).

### Detection and quantification of cell apoptosis

Living, apoptotic and necrotic cells were detected and quantified by fluorescence microscopy and using Hoechst staining (Beyotime, Nanjing). After treatment, H9c2 cells were then harvested, washed twice with ice‐cold PBS, and evaluated for apoptosis by double staining with FITC conjugated Annexin V and propidium iodide (PI) in binding buffer for 30 min. using a FACSCalibur flow cytometer (BD Biosciences).

### Animals and treatment

The animals were obtained from Animal Center of Wenzhou Medical University. All animal care and experimental procedures complied with the ‘Ordinance in Experimental Animal Management’ (Order NO. 1998‐02, Ministry of Science and Technology, China) and were approved by the Wenzhou Medical College Animal Policy and Welfare Committee (Approval Document NO. wydw2014‐0105).

Twenty‐one male Wistar rats (360–370 g) were randomly divided into three weight‐matched groups. Seven rats were fed with low‐fat diet (cat. #MD12031; MediScience Diets Co. Ltd, Yangzhou, China) containing 10 kcal% fat, 20 kcal% protein and 70 kcal% carbohydrate for 12 weeks, and served as a normal control group (Ctrl). Remaining 14 rats were fed with HFD (cat. #MD12033; MediScience Diets Co. Ltd) containing 60 kcal% fat, 20 kcal% protein and 20 kcal% carbohydrate for 12 weeks. After 8 weeks of feeding, HFD‐fed rats were further divided into two groups: HFD group (*n* = 7) and HFD plus X22‐treated group (*n* = 7). X22 was given daily by oral gavage at a dose of 20 mg/kg, respectively, in 0.5% CMCNa solution for 4 weeks. Rats in the Ctrl and HFD group were gavaged with vehicle only. All the animals were provided with food and water *ad libitum*. In the experiment process, bodyweight and blood glucose were monitored once every week. At the end of experimental period, all the animals were killed by cervical decapitation. The bodyweight was recorded and blood samples were collected and centrifuged at 4°C for 10 min. to collect serum. The heart was excised aseptically, blotted dry and the weight was recorded followed by immediate freezing in liquid nitrogen and then stored at −80°C before further analysis.

### Histological and histochemical analyses

Excised heart tissue specimens were fixed in 4% formalin processed in graded alcohol, xylene, and then embedded in paraffin. Paraffin blocks were sliced into sections of 5 μm in thickness. After rehydration, the sections were stained with haematoxylin and eosin, Masson's trichrome or sirius red, respectively. Each image of the sections was captured using a light microscope (400× amplification/image; Nikon).

### Immunohistochemical determination

The paraffin samples (5 μm) were removed from the sections with xylene, rehydrated in graded alcohol series, subjected to antigen retrieval in 0.01 mol/l citrate buffer (pH 6.0) through use of microwave and then placed in 3% hydrogen peroxide in methanol for 30 min. at room temperature. After blocking with 5% BSA, the sections were incubated with anti‐3‐nitrotyrosine (NT) antibody (1:500), anti‐TNF‐α antibody (1:500) or anti‐cluster of differentiation (CD68) (1:200) overnight at 4°C, followed by the secondary antibody (1:200; Santa Cruz Biotechnology). The reaction was visualized with 3,3′‐diaminobenzidine solution. After counterstaining with haematoxylin, the sections were dehydrated and viewed under fluorescence microscope (400× amplification; Nikon).

### Measurements of the level of serum lipid

The components of serum lipid including the total triglyceride (TG), total cholesterol (TCH) and low‐density lipoprotein (LDL) were detected using commercial kits (Nanjing Jiancheng Bioengineering Institute, Jiangsu, China).

### Real‐time quantitative PCR

Total RNA was isolated from tissues (50–100 mg) or cells using TRIZOL (Life Technologies, Carlsbad, CA, USA). Reverse transcription and quantitative PCR (RT‐qPCR) were performed using MMLV Platinum RT‐qPCR Kit (Life Technologies). Real‐time qPCR was carried out using the Eppendorf MasterCycler RealPlex 4 instrument (Eppendorf, Hamburg, Germany). Primers for genes including TNF‐α, IL‐6, IL‐1β, intercellular adhesion molecule 1 (ICAM‐1), vascular cell adhesion molecule 1 (VCAM‐1), connective tissue growth factor (CTGF), Collagen I, nuclear factor (erythroid‐derived 2)‐like (Nrf2), heme oxygenase‐1 (HO‐1), glutamate‐cysteine ligase catalytic subunit (GCLC), glutamate‐cysteine ligase modifier subunit (GCLM), ANP, B‐type natriuretic peptide (BNP) and β‐actin were obtained from Life Technologies. The primer sequences used are shown in Table S1. The relative amount of each gene was normalized using β‐actin as an internal control.

### Western blot assay

Tissues (30–50 mg) or cells were lysed, and protein concentrations were determined by using the Bradford protein assay kit (Bio‐Rad). Aliquots (about 100 μg cellular protein) were subjected to electrophoresis and transferred to nitrocellulose membranes, which were then blocked in Tris‐buffered saline, containing 0.05% Tween 20 and 5% non‐fat milk. The polyvinylidene fluoride membrane was then incubated overnight with specific antibodies. Following incubation with appropriate secondary antibodies, immunoreactive proteins were visualized with ECL (Bio‐Rad) reagent and quantitated by densitometry. Stripped membranes were reprobed with antibodies for glyceraldehyde 3‐phosphate dehydrogenase, to assess protein loading. The amounts of the proteins were analysed using Image J analysis software version 1.38e and normalized to their respective control.

### Statistical analysis

All *in vitro* experiments were assayed in triplicate repeat. Data are expressed as mean ± S.E.M. All statistical analyses were performed using GraphPad Pro, Prism 5.0 (GraphPad, San Diego, CA, USA). Student's *t*‐test and two‐way anova were employed to analyse the differences between sets of data. A *P*‐value <0.05 was considered significant.

## Results

### X22 mitigated NF‐κB‐mediated inflammatory response in PA‐induced H9c2 cells

The chemical structure of X22 is shown in Figure 1A. To determine whether X22 exhibits anti‐inflammatory activity in FFA‐stimulated cells, an immunofluorescence assay was conducted to detect the expression and distribution of NF‐κB p65 in H9c2 cells. Our results showed that in PA‐treated cells, NF‐κB p65 accumulated in the nuclei (Fig. 1B). Further protein analysis confirmed this translocation of p65 from the cytoplasm to nucleus, showing that in PA‐treated cells, there was a decrease of p65 in the cytoplasm, coupled with an increase of p65 in the nucleus (Fig. 1C). Since the degradation of inhibitor of NF‐κB (IκB‐α) is an important component in mediating the activation of the NF‐κB pathway, we assayed for IκB‐α levels. While PA induced IκB‐α protein degradation, pre‐treatment with X22 restored the protein level of IκB‐α back to normal media levels (Fig. 1D). Building on these results, we examined the mRNA expression of NF‐κB‐activated inflammatory cytokines, such as TNF‐α, IL‐1β and IL‐6, and cell adhesion molecules, ICAM‐1 and VCAM‐1. As shown in Figure 1E and F, we found that treatment with X22 significantly inhibited PA‐induced mRNA expression of both inflammatory cytokines, TNF‐α, IL‐1β and IL‐6, and adhesion molecules, ICAM‐1 and VCAM‐1.

### X22 prevents PA‐induced ROS production and oxidative stress in H9c2 cells

**Figure 2:**
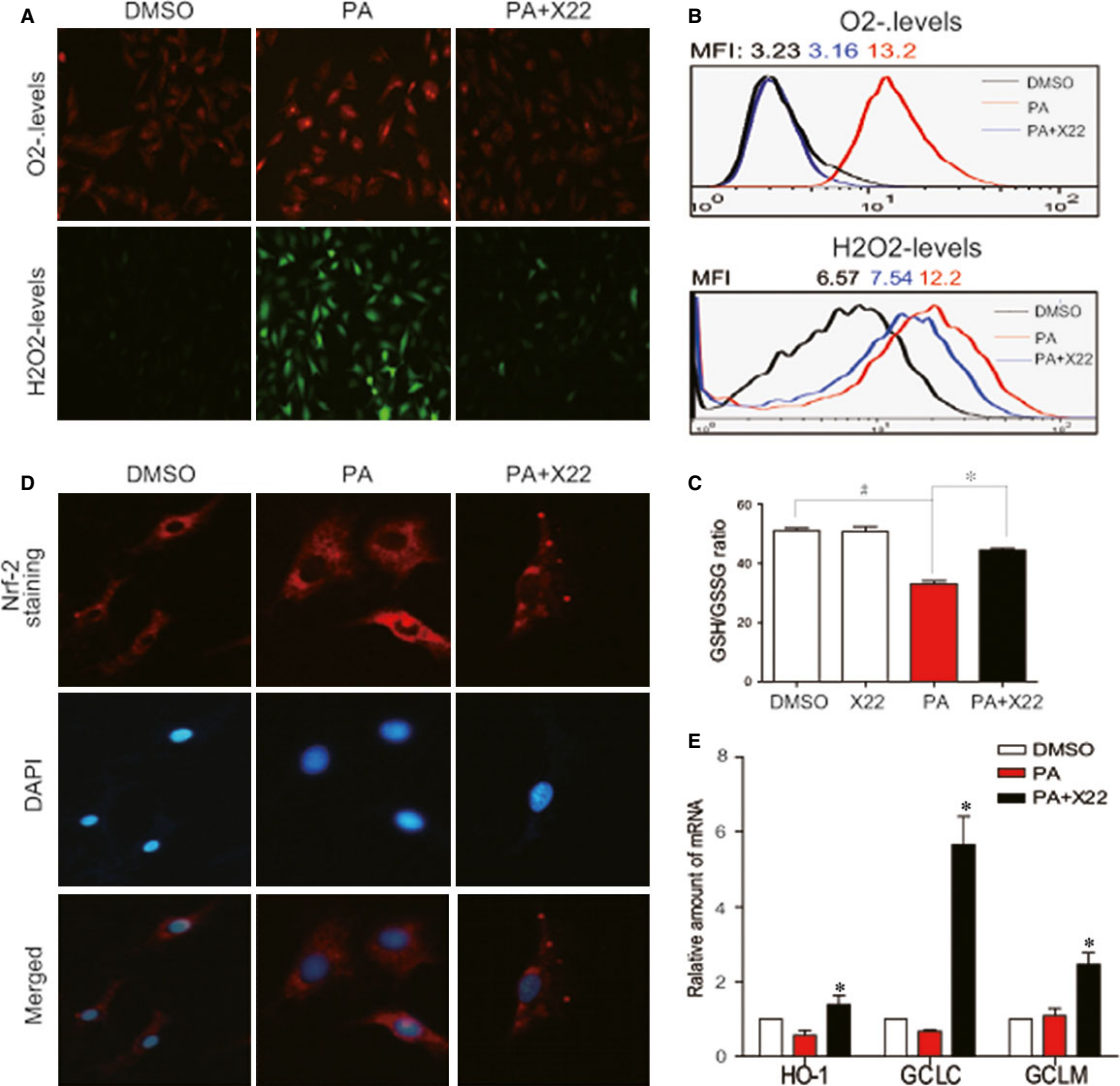
X22 attenuated PA‐induced myocardial oxidative stress in H9C2 cells. H9c2 cells were pretreated with X22 (20 μM) for 1 hr and then incubated with PA (500 μM) for 10 hrs. (**A**) DHE and DCFH‐DA probes were loaded, respectively, and the ROS positive cells were detected using fluorescence microscope. The images represent three independent experiments. (**B**) After being loaded with the probes, cells were processed by flow cytometry analysis for H_2_O_2_ levels, and the data are represented by mean fluorescence intensity (MFI) value. (**C**) After treatment, cells were lysed and the 30 μl of collected proteins were performed to GSH/GSSG assay. (**D**) X22 activates Nrf2. H9c2 cells were pretreated with X22 (20 μM) for 1 hr and then incubated with PA (500 μM) for 6 hrs. Then, the nuclear translocation of Nrf‐2 was detected using fluorescence microscope. (**E**) Cells were collected and the total RNA was extracted and processed for real‐time RT‐qPCR assay for Nrf2 downstream genes including HO‐1, GCLC and GCLM (*n* = 3 for all *in vitro* experiment; ^#^ and *P < 0.05).

Previous studies have shown that elevated FFA levels can lead to increased oxidative stress in cardiovascular tissues 22. It has also been shown that at a concentration of 500 μM, PA can stimulate ROS production and increase oxidative stress in H9c2 1. We wanted to examine if X22 could prevent PA‐induced ROS production and oxidative stress in H9c2 cells. Using DHE (for O_2_^−^) and DCFH‐HA (for H_2_O_2_^−^) probes, we were able to show that ROS production was significantly increased following PA treatment (500 μM for 10 hrs). However, pre‐treatment with X22 at 20 μM for 1 hr significantly decreased ROS production back to control culture levels (Fig. 2A). The mean fluorescent intensity values also showed that X22 significantly reduced PA‐induced increases in ROS‐positive cells (Fig. 2B). In addition, we used GSH/GSSG probe to confirm antioxidant activity of X22. The result in Figure 2C showed that X22 reversed PA‐decreased GSH/GSSG ratio in H9c2 cells. We also wanted to confirm if X22′s observed antioxidant properties were through the modulation of the anti‐oxidant Nrf2. Using fluorescent stain, we observed that H9c2 cells treated with PA and X22 showed an increase in the nuclear translocation of Nrf2, indicating X22 could promote Nrf2 transcriptional activity (Fig. 2D). Furthermore, when we examined the downstream target genes of Nrf2, including HO‐1, GCLC and GCLM, it was observed that X22 significantly induced the mRNA expression of these anti‐oxidant genes (Fig. 2E).

### X22 attenuates PA‐induced cardiac hypertrophy and fibrosis in H9c2 cells

**Figure 3:**
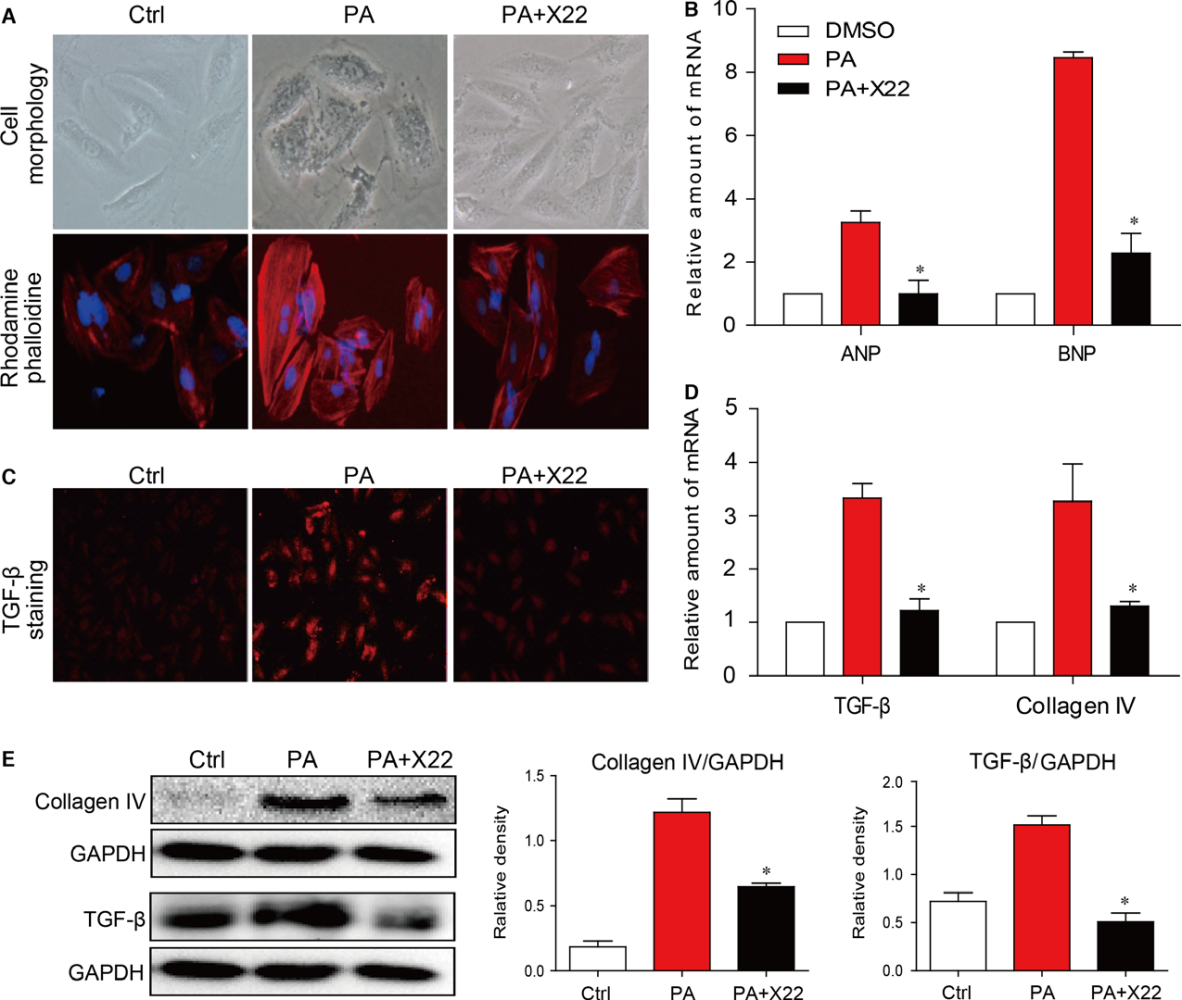
X22 attenuated PA‐induced cardiac hypertrophy and fibrosis in H9C2 cells. (**A** and **B**) X22 reduces hypertrophy. H9c2 cells were pretreated with X22 (20 μM) for 1 hr and then incubated with PA (500 μM) for 6 hrs. (**A**) Representative images for cell morphology analysis were obtained using light microscopy and rhodamine‐phalloidin/DAPI immunofluorescence staining; *n* = 3 separate determinations. (**B**) Real‐time RT‐qPCR analysis for pro‐hypertrophic genes expression. (**C**–**E**) X22 reduces fibrosis. H9c2 cells were pretreated with X22 (20 μM) for 1 hr and then incubated with PA (500 μM) for 12 hrs. (**C**) Immunofluorescence staining for TGF‐β in the cells was performed as described in ‘Materials and methods’; *n* = 3 separate determinations. (**D**) Real‐time RT‐qPCR analysis for pro‐fibrotic genes expression. (**E**) Shown are representative Western blot analysis for expression of pro‐fibrosis proteins; *n* = 3 separate determinations. For real‐time qPCR assay, data are reported as the mean ± S.E. (*P < 0.05; *n* = 4 of all experiments).

The effect of X22 on cardiac cell hypertrophy was examined by using rhodamine‐phalloidin staining. As referred to the literature 1, the H9c2 cells were pretreated with X22 at 20 μM for 1 hr and then incubated with PA at 500 μM for 6 hrs. As shown in Figure 3A, X22 significantly suppressed PA‐induced hypertrophy in H9c2 cells. X22 also significantly inhibited PA‐induced mRNA expression of ANP and BNP (Fig. 3B). The effect of X22 on fibrosis was determined using TGF‐β staining, which revealed that X22 also significantly reduced PA‐induced increase in TGF‐β (Fig. 3C). Further, RT‐qPCR analysis and Western blot analysis showed that X22 suppressed PA‐induced mRNA expression of TGF‐β, CTGF and collagen I (Fig. 3D), as well as protein expression of collagen IV and TGF‐β (Fig. 3E).

### X22 protects against PA‐induced apoptosis in H9c2 cells

**Figure 4:**
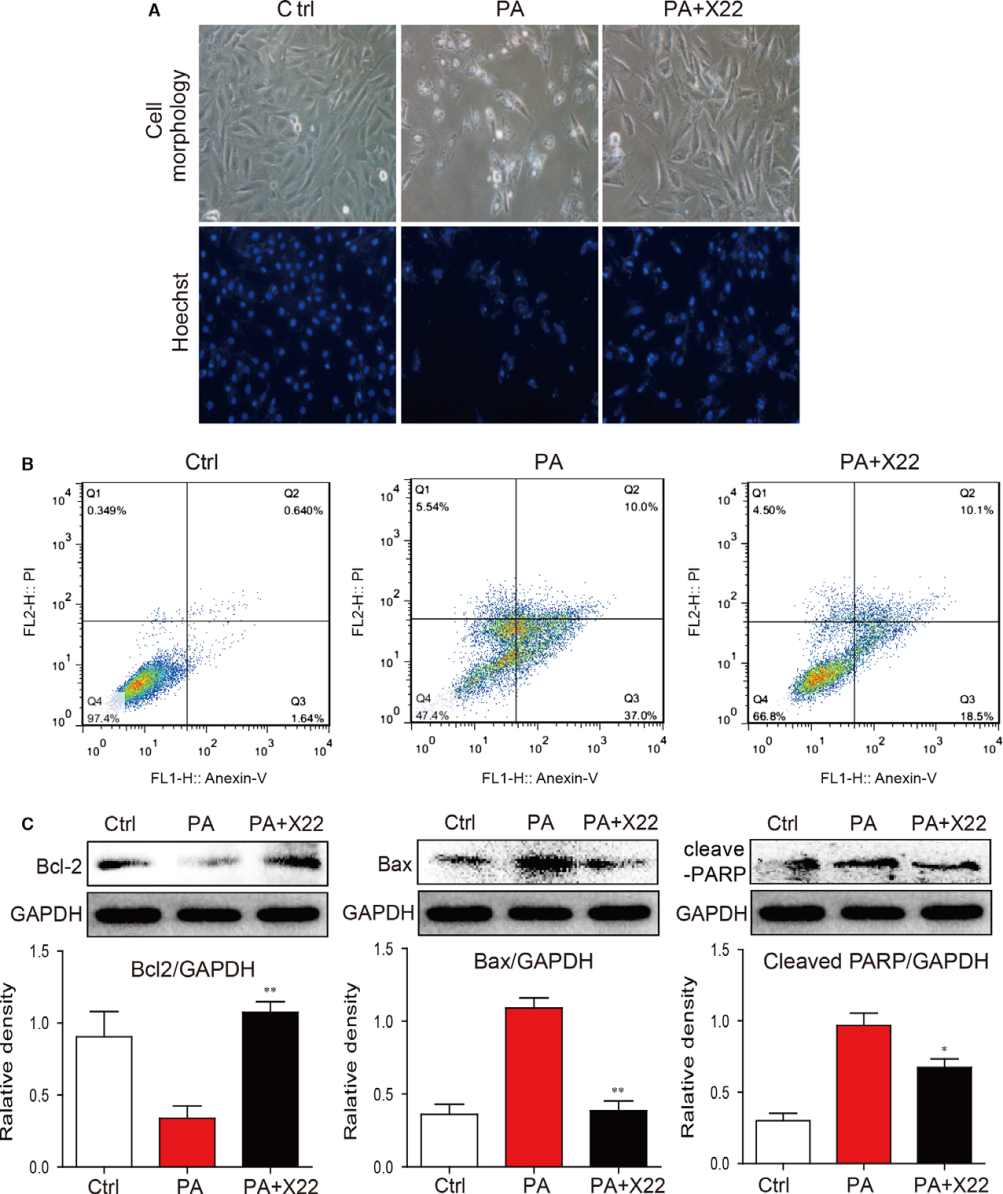
X22 attenuated PA‐induced cell apoptosis in H9c2 cells. H9c2 cells were pretreated with X22 (20 μM) for 1 hr and then incubated with PA (500 μM) for 18 hrs. (**A**) Representative images for cell morphology analysis were obtained using light microscopy and Hoechst immunofluorescence staining. (**B**) Representative plots of flow cytometry with annexinV/PI. (**C**) The Western blot analysis for apoptotic proteins expression of Bax, Bcl‐2 and cleaved‐PARP in H9c2 cells (*n* = 3 for each experiment; *P < 0.05, **P < 0.01).

We further determined the protective effect of X22 on PA‐induced apoptosis in H9c2 cells. H9c2 cells were pretreated with X22 at 20 μM for 1 hr and then incubated with PA at 500 μM for 18 hrs. As shown in Figure 4A, PA induced apoptosis in H9c2 cells, with significant morphological changes and decreased Hoechst staining of cells. An assay for annexin V/PI confirmed these results showing that the relative percentage of apoptotic cells and the number of annexin V positive cells were significantly higher in PA‐treated cells (Fig. 4B). However, in cells pre‐treated with X22, cell morphological changes were less significant with decreased number of apoptotic cells, as evidenced by both Hoechst staining and flow cytometry (Fig. 4A and B). Furthermore, when we examined the protein levels of key proteins involved in the apoptotic pathway, such as B‐cell lymphoma (Bcl)‐2, Bcl‐2‐associated X protein (Bax) and PARP, we observed significant PA‐induced increases in the protein levels of pro‐apoptotic proteins Bax and cleaved PARP and decreased levels of Bcl‐2 (Fig. 4C). Treatment with X22 reversed these changes, suppressing PA‐induced increases in protein expression of Bax and cleaved PARP and inducing protein expression of Bcl‐2 (Fig. 4C). In addition, X22‐alone treatment did not affect NF‐κB p65 nuclear translocation, ROS level, SOD activity, hypertrophy and collagen‐4 and cleaved PARP expression (Fig. S1) in H9c2 cells, indicating no difference between Ctrl group and X22‐alone group.

### Administration of X22 attenuated HFD‐induced changes in the lipid profiles of rats

**Figure 5:**
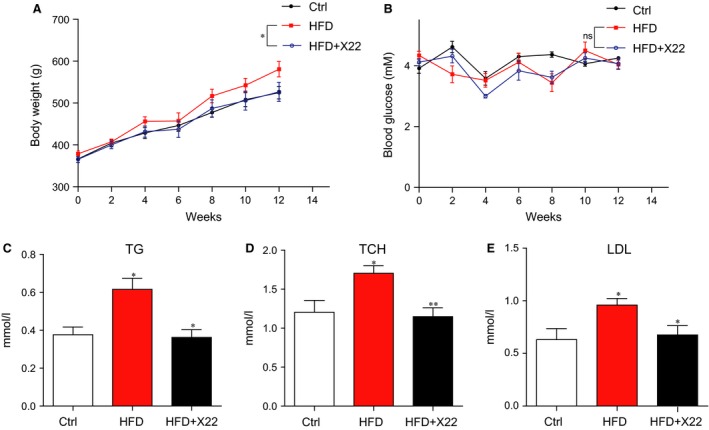
The effects of X22 on bodyweight, blood glucose and blood lipid profile in HFD rats. Male rats weighing 360–370 g were first fed either a normal diet (Ctrl) or high‐fat diet (HFD) for 8 weeks. The HFD rats were subsequently split into two groups, one group treated with X22 (20 mg/kg) for 4 weeks and the other group with vehicle control (*n* = 7 in each group). The bodyweight and blood glucose were monitored weekly. At the end of experiment, the rats were killed, the blood samples were collected and centrifuged, and serum was isolated. (**A**) The bodyweight. (**B**) The serum glucose level. (**C**–**E**) The serum lipid profile, including triglycerides (TG), total cholesterol (TCH), low‐density lipoprotein (LDL) (*P < 0.05, **P < 0.01, *n* = 7 in each group).

We used HFD‐fed rat model to investigate whether X22 exhibits cardioprotective effects *in vivo* in obesity. Rats on a HFD for 8 weeks were subsequently treated with X22 at a dosage of 20 mg/kg/day or vehicle control for 4 weeks. Rats fed a normal diet (ND) were used as the control group. The bodyweight and blood glucose levels were monitored every week, and at the end of the experiment, the blood samples were collected and serum was analysed for levels of TG, TCH and LDL. High‐fat diet‐fed rats became slightly obese with a bodyweight above 550 g on average at the 12‐week time‐point, while treatment with X22 markedly reduced the bodyweight gain in HFD‐fed rats (*P* < 0.05 *versus* HFD group, Fig. 5A). Both HFD‐fed rats and X22‐treat HFD rats did not exhibit significant changes in the level of blood glucose when compared with the ND group (Fig. 5B). Compared with the ND‐fed rats, the HFD‐fed rats displayed significantly elevated serum levels of TG, TCH and LDL (Fig. 5C–E). In contrast, treatment with X22 significantly inhibited HFD‐induced increases of TG, TCH and LDL. These data indicate that X22 also possesses anti‐obesity effects in HFD‐fed rats.

### X22 attenuated HFD‐induced inflammation in the myocardial tissues of HFD‐fed rats

**Figure 6:**
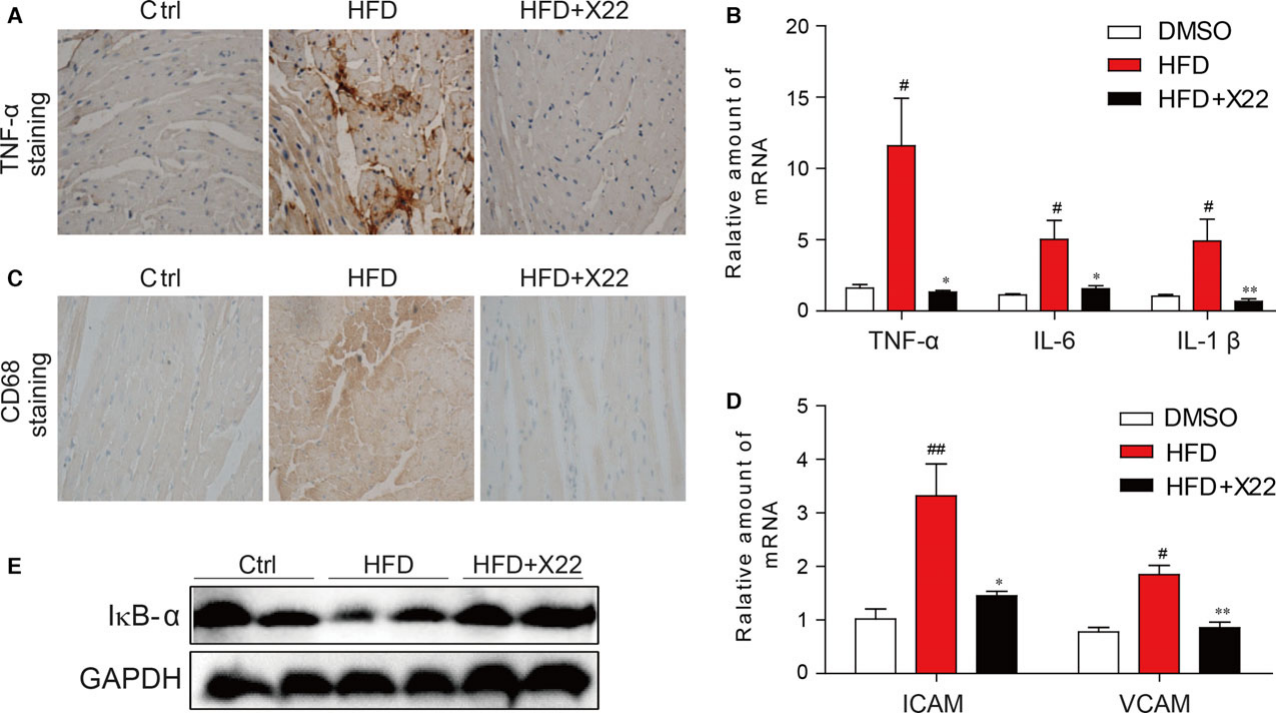
X22 attenuated HFD‐induced inflammation in HFD‐fed rats. (**A**) Representative images for immunohistochemical staining for TNF‐α accumulation in the formalin‐fixed myocardial tissues (400× magnification). (**B**) The mRNA expression of the inflammation markers IL‐6, IL‐1β and TNF‐α in the myocardial tissues. (**C**) Representative images for immunohistochemical staining for CD68 accumulation in the formalin‐fixed myocardial tissues (400× magnification). (**D**) The mRNA expression of the adhesion markers VCAM‐1 and ICAM‐1 in the myocardial tissues. ICAM‐1: intercellular adhesion molecule‐1; VCAM‐1: vascular cell adhesion molecule‐1. (**E**) The Western blot analysis for the protein expression of IκB‐α in the myocardial tissues (#, *versus* DMSO samples; *, *versus* HFD samples; ^#^ and *P < 0.05, ^##^ and **P < 0.01; *n* = 7 per group).

The inflammatory and oxidative indexes were determined in the myocardial tissues of HFD‐fed rats. Immunohistochemical staining for TNF‐α accumulation in formalin‐fixed myocardial tissues in HFD‐fed rats showed a significant increase in the accumulation of TNF‐α (Fig. 6A). This result was further supported through RT‐qPCR analysis, which revealed a significant increase in the mRNA expression of inflammatory markers TNF‐α, IL‐6 and IL‐1β (Fig. 6B). In contrast, treatment with X22 normalized TNF‐α protein level (Fig. 6A) and significantly inhibited HFD‐induced inflammatory cytokine expression (Fig. 6B). In addition, X22′s anti‐inflammatory properties were also confirmed by determining adhesion molecule expression using immunohistochemical staining for CD68, a marker of infiltrated macrophages. CD68 staining revealed a significant accumulation of CD68 in the myocardial tissues of HFD‐fed rats that was normalized following treatment with X22 (Fig. 6C). Furthermore, while HFD‐induced marked increases in the mRNA expression of adhesion markers, VCAM‐1 and ICAM‐1, in myocardial tissues, X22 administration significantly inhibited HFD‐induced expression of both adhesion markers (Fig. 6D). We also assessed whether X22′s effect on the expression of inflammatory cytokines was due to changes in the NF‐κB pathway. As shown in Figure 6E, while protein levels of IκB‐α were significantly decreased in the HFD group, indicating NF‐κB activation, treatment with X22 restored the IκB‐α to ND levels.

### X22 attenuated HFD‐induced oxidative stress in the myocardial tissues of HFD‐fed rats

**Figure 7:**
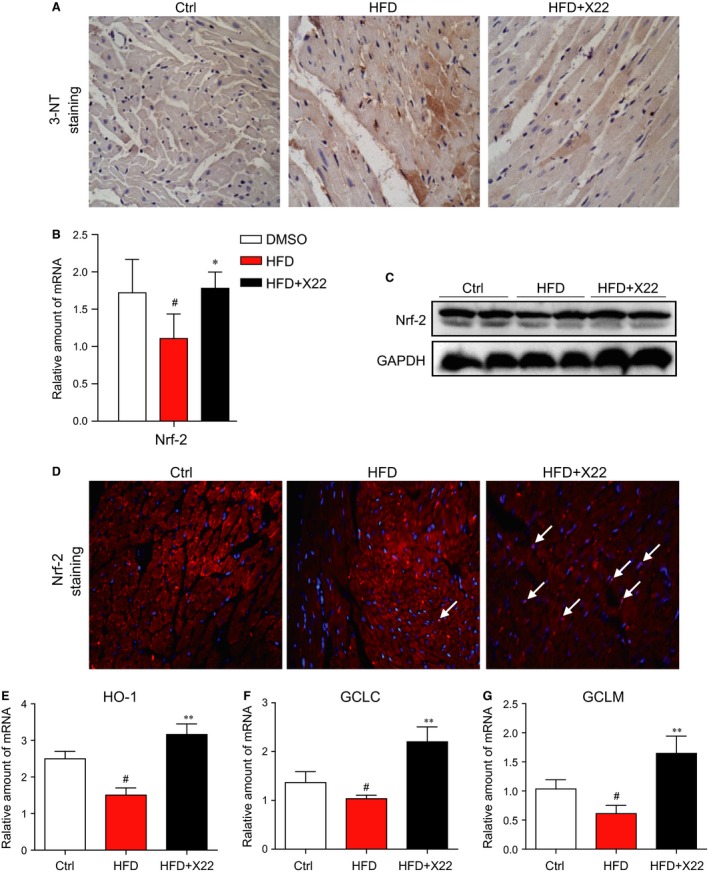
X22 attenuated HFD‐induced myocardial oxidative stress in HFD‐fed rats. (**A**) Representative images for immunohistochemical staining of 3‐NT accumulation in the formalin‐fixed myocardial tissues (400× magnification). (**B**) The mRNA expression of the anti‐oxidative markers Nrf‐2 in the myocardial tissues was determined by real‐time qPCR assay. (**C**) The Western blot analysis for the protein expression of Nrf‐2 in the myocardial tissues. (**D**) Representative images for immunohistochemical staining of Nrf2 and DAPI in the formalin‐fixed myocardial tissues (200× magnification). (**E**–**G**) The mRNA expression of the anti‐oxidative genes HO‐1, GCLC and GCLM in the heart tissues was determined by real‐time qPCR assay (*, *versus* HFD group; ^#^, *versus* Ctrl group; * and ^#^ *P* < 0.05; ** and ^##^ *P* < 0.01; *n* = 7 per group).

We then examined the effects of X22 in HFD‐induced oxidative stress in rat myocardial tissues. 3‐NT was used as a biomarker for formation of ROS and reactive nitrogen species (RNS). Staining for the accumulation of 3‐NT in HFD‐fed rats revealed that HFD led to a significant increase of 3‐NT, indicative of increased ROS/RNS accumulation, which was normalized with treatment of X22 (Fig. 7A). Data from Figure 7B and C also showed that Nrf2 mRNA and protein expression was significantly decreased in the myocardial tissue of HFD‐fed rats. Treatment with X22 induced a significant increase in the mRNA and protein expression of Nrf‐2 (Fig. 7B and C). Fluorescent staining assay also showed that X22‐treated mouse hearts have the increased nuclear distribution of Nrf2 when compared with HFD‐alone group, indicating a transcriptional activation of Nrf2 by X22 (Fig. 7D). Furthermore, we also examined the downstream target genes of Nrf2, including HO‐1, GCLC and GCLM by real‐time qPCR assay. As expected, it was observed that X22 significantly increased the mRNA levels of these anti‐oxidant genes in HFD‐fed mouse hearts (Fig. 7E–G).

### X22 administration improved cell histological abnormalities, hypertrophy, fibrosis and apoptosis in the myocardial tissues of HFD‐fed rats

**Figure 8:**
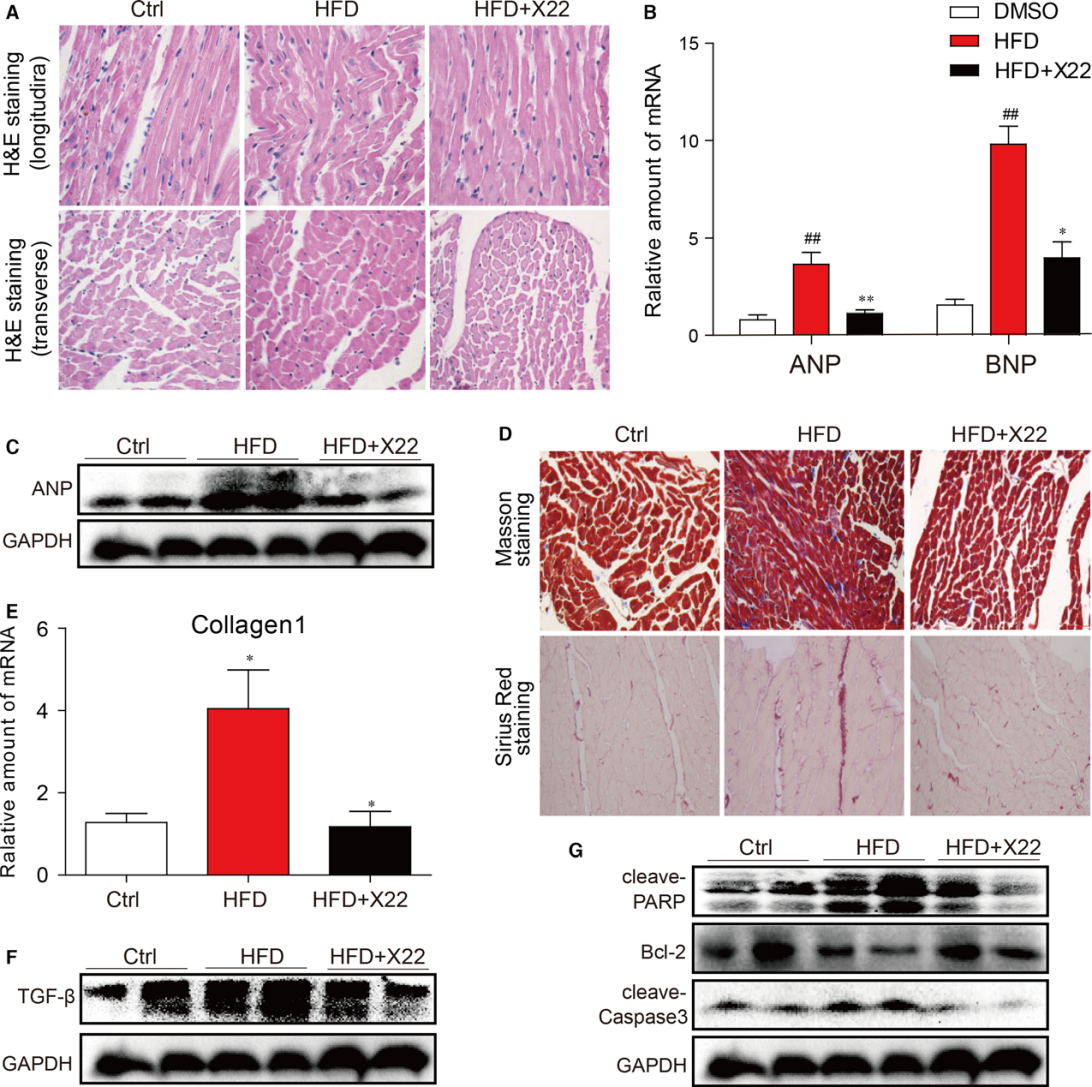
X22 decreased HFD‐induced cardiac remodelling (**A**–**C**), fibrosis (**D**–**F**) and apoptosis (**G**) in HFD‐fed rats. (**A**) Representative images for the haematoxylin and eosin staining in the formalin‐fixed myocardial tissues (400× magnification). (**B**) The mRNA expression of the hypertrophic markers ANP and BNP in the myocardial tissues. (**C**) The Western blot analysis for the protein expression of ANP in the myocardial tissues. (**D**) Representative images for the Masson staining and Sirius Red staining in the formalin‐fixed myocardial tissues (400× magnification). (**E**) The mRNA expression of the fibrosis marker collagen I in the myocardial tissues. (**F**) The Western blot analysis for the protein expression of TGF‐β in the myocardial tissues. (**G**) The Western blot analysis for the protein expression of cleaved‐PARP, cleaved‐caspase3 and Bcl‐2 in the myocardial tissues (#, *versus* DMSO samples; *, *versus* HFD samples; ^#^ and *P < 0.05, ^##^ and **P < 0.01, *n* = 7 per group).

To further investigate the *in vivo* cardioprotective effects of X22, we examined X22 effects on the morphology of the heart. Haematoxylin and eosin staining showed that hearts of HFD‐fed rats displayed structural abnormalities, including broken fibres and irregular cellular structures, and significantly increased cardiomyocyte transverse cross‐sectional area, while those of HFD‐fed rats treated with X22 did not (Fig. 8A). Furthermore, in the cardiac tissues of HFD‐fed rats, cardiac hypertrophy was characterized with increased cell surface area, increased mRNA expression of cardiac hypertrophic markers, ANP and BNP (Fig. 8B), and increased protein expression of ANP (Fig. 8C). However, as shown in Figure 8A–C, X22 treatment had a protective effect in HFD‐induced cardiac remodelling and also significantly inhibited the mRNA expression of ANP and BNP, suggesting that X22 prevents the development of cardiac hypertrophy in HFD‐fed rats.

Further staining with Masson's stain and Sirius red demonstrated the anti‐fibrotic properties of X22 *in vivo*. While Masson's and Sirius red staining revealed a significant increase in collagen accumulation and fibrosis in the hearts of HFD‐fed rats, treatment with X22 markedly reduced the degree of collagen deposition and fibrosis (Fig. 8D). These observations were further confirmed through RT‐qPCR analysis, which revealed increases in the mRNA expression of type 1 collagen (Fig. 8E) and protein expression of cardiac fibrosis marker TGF‐β in HFD‐fed rats (Fig. 8F). These changes were not observed and significantly blocked by administration of X22. Western blot analysis for protein expression of key apoptotic proteins showed that HFD‐fed rats had decreased levels of Bcl‐2, a key regulator of apoptotic proteins, and increased levels of both cleaved PARP and cleaved‐caspase 3 (Fig. 8G). In contrast, HFD‐fed rats treated with X22 showed significantly increased protein levels of Bcl‐2 and reduced levels of cleaved PARP and cleaved‐caspase 3, suggesting that X22 has the anti‐apoptotic properties.

## Discussion

Recent studies have implicated chronic inflammation and oxidative stress in the pathophysiology of obesity‐related cardiovascular disorder 23, 24, 25, 26. Increased and uncontrolled production of inflammatory cytokines and reactive oxygen species due to hyperlipidemia impairs regular cellular function and causes cell apoptosis in an array of tissues, including the heart 27. Therefore, due to the potential roles inflammation and oxidative stress play in cardiovascular disorders, molecules with anti‐inflammatory and antioxidant properties may be targets to enhance the efficacy of therapeutic options for obesity and HFD‐induced cardiovascular disorders.

A number of studies have demonstrated that imidazopyridines have a wide variety of pharmacological activities, such as anti‐inflammatory 17, 28, antioxidant 20, antiviral 29 and anticancer 21, 22. In a previous study, our group synthesized imidazopyridine derivatives and evaluated them on the anti‐inflammatory activity. The results from this study showed that a imidazopyridine derivative X22 does inhibit PA/HFD‐induced inflammatory response, oxidative stress and apoptosis *in vitro* in rat H9c2 cells and *in vivo* in HFD‐fed rats. At the same time, X22 treatment significantly decreased the hyperlipidemia profile (TG, TCH and LDL) and also improved cell histological abnormalities, hypertrophy and fibrosis in myocardial tissues of HFD‐fed rats, suggesting that X22 treatment has a protective effect on HFD‐induced cardiac remodelling and injury.

Hyperlipidemia is defined as a condition with elevated levels of cholesterol, triglycerides, LDL and FFAs, which have been shown to increase the risk of heart disease, stroke and other health problems. In obesity, FFA levels are usually elevated, and prolonged and chronic elevation can result many pathophysiological consequences. Therefore, FFAs play an important role both in the development of obesity‐related complications and atherosclerotic vascular diseases 30. Palmitic acid is a major saturated FFA in the plasma that stimulates inflammatory cytokine expression and ROS production both in cultured aortic smooth muscle cells and endothelial cells 31. In this study, we also observed a significant increase in the production of inflammatory cytokines and of ROS and oxidative stress in H9c2 cells treated with PA (Figs 1 and 2). Furthermore, PA has also been shown to affect vascular functions 32, and we also observed PA‐induced cardiac hypertrophy and fibrosis in H9c2 cells (Fig. 3).

The relationship between obesity and inflammation has been well‐established. Obesity is now often associated with a state of chronic, low‐grade inflammation, suggesting that inflammation may serve as a potential mechanism resulting in obesity‐related, cardiovascular complications 33, 34. Elevated FFA levels have also been linked to increased production of pro‐inflammatory cytokines 1, 23, 24, 25, 26, 34, 35, and in macrophages, FFAs have been found to trigger inflammatory responses *via* toll‐like receptor 4. The NF‐κB pathway is critical in the regulation of inflammatory responses with many of its downstream targets being inflammatory cytokines, chemokines, cell adhesion molecules, stress response genes and regulators of apoptosis. Our results demonstrated that PA/HFD decreased IkB‐α level and increased p65 translocation and NF‐κB activity *in vitro* in H9c2 cardiac cells (Fig. 1) and *in vivo* in the myocardial tissue of rats (Fig. 6), resulting in increased expression of pro‐inflammatory cytokines TNF‐α, IL‐6 and IL‐1β and cell adhesion molecules VCAM‐1 and ICAM‐1. In contrast, X22 significantly inhibited NF‐κB activation, attenuating against PA/HFD‐induced expression of inflammatory cytokines and cell adhesion molecules. These results indicate that X22′s inhibits PA/HFD‐induced inflammation *via* upregulation of IkB‐α and subsequent inactivation of NF‐κB.

While we had previously discovered that X22 had anti‐inflammatory properties, it was through this study that we explored its potential antioxidant properties. However, the connection between inflammation and oxidative stress with regard to obesity has been well documented. Adipocytes and pre‐adipocytes have been identified as sources of pro‐inflammatory cytokine production, including TNF‐α, IL‐1β and IL‐6, and these cytokines are also potent stimulators for the production of reactive oxygen and nitrogen species by macrophages and monocytes 23. Therefore, the increased presence of excessive adipose tissue, which leads to a rise in production of inflammatory cytokines, may be responsible for elevated levels of ROS and subsequent oxidative stress. Furthermore, many genes involved in oxidative stress have been confirmed to be either directly or indirectly regulated by Nrf2 1. Under normal conditions, Nrf2 is found within the cytoplasm, but under stressed conditions, activated Nrf2 travels to the nucleus where it binds to the promoter regions of anti‐oxidative genes, initiating the expression of those genes and subsequent proteins 36. Others have also demonstrated that Nrf2 inhibits the oxidative stress in liver induced by FFA accumulation in HFD‐fed mice 37, plays a role in pulmonary protection 38 and is critical in defence against high glucose‐induced oxidative damage in cardiomyocytes 1, 39. The results from this study confirmed these findings. As shown in Figures 2 and 7, both HFD and PA activated Nrf2 expression and nuclear translocation. Furthermore, we found that X22 could reverse these changes, leading to an increased expression of Nrf2 and Nrf2‐downstream genes in HFD‐fed rats and reduced translocation of Nrf2 into the nucleus of PA‐treated H9c2 cells. These results confirmed X22′s antioxidant properties and that X22′s observed protective effects against oxidative stress‐induced cardiac injury could be potentially due to its regulation and activation of Nrf2.

Oxidative stress and inflammation in cells are strongly associated with cell apoptosis 40, 41. As a result of PA/HFD‐induced oxidative stress and inflammation, we showed that cardiac cells undergo apoptosis (Figs 4 and 8G). We also investigated the protective effects of X22 against PA/HFD‐induced changes in cell morphology and apoptosis, and our findings show that X22 inhibited PA/HFD‐induced cardiomyocyte apoptosis. In HFD‐fed rats, X22‐treatment resulted in decreased levels of cleaved PARP and cleaved‐caspase 3 and increased Bcl‐2 levels (Fig. 8G). From these results, we find it reasonable to conjecture that X22 attenuates PA/HFD‐induced apoptosis by inhibiting the NF‐κB pathway and alleviating oxidative stress. Oxidative stress and inflammation have also been implicated in the development and progression of cardiac hypertrophy 42, 43. Increased expression of proteins such as ANP and BNP and increased cell surface area are often seen as important molecular markers for cardiac hypertrophy. Our results showed that while PA and HFD led to a significant increase in the gene expression of ANP and BNP both *in vitro* and *in vivo* and decrease cell surface area *in vivo* (Figs 3 and 8), X22 was able attenuate the PA/HFD‐induced cardiac hypertrophy. Another key feature of cardiac remodelling is fibrosis, which is characterized by the expansion of the extracellular matrix as a result of collagen accumulation. Inflammation and oxidative stress also contribute to cardiac fibrosis 44. Oxidative stress was found to either directly or indirectly affect the progression of cardiac fibrosis through the activation of TGF‐β 45. As expected, we observed that while PA and HFD resulted in increased expression of pro‐fibrotic markers, such as collagen I, TGF‐β and CTGF (*in vitro*). X22 significantly inhibited this increased expression and produced visible changes in the cardiac tissues of HFD‐fed rats, proving the anti‐fibrotic activity of X22 in the cardiac tissue of HFD‐fed rats.

The oxidative stress and inflammatory pathways in obesity‐related cardiomyopathy are closely interrelated. They interact and crosslink throughout the complicated process of obesity‐related cardiomyopathy. Thus, independently blocking either pathway may be not effective for the treatment of this disease. Our results further suggest the tremendous therapeutic potential in treating obesity‐related cardiovascular complications by attenuating both the initial oxidative stress and inflammation induced by hyperlipidemia. Agents including X22 with both anti‐oxidant and anti‐inflammatory properties may attract more attention for the treatment of this disease.

In addition, it should be noted that treatment with X22 resulted in decreased bodyweight gain and serum TG, TCH and LDL levels in HFD‐fed rats (Fig. 5A), indicating that the obesity‐lowering effects of X22 are also significant. Thus, the *in vivo* cardioprotective effects of X22 also resulted from, at least partly, its obesity‐lowering action. From the animal outcome, we cannot differentiate between the direct effects of X22 as an anti‐inflammatory and anti‐oxidative agent against hyperlipidemia‐induced cardiac injuries and the secondary effects of X22 as an‐obesity agent for re‐establishment of a healthy lipid profile. Despite the *in vitro* data confirmed the anti‐oxidant and anti‐inflammatory properties of X22, the integrated merits of X22 may result in the cardioprotective effects and make X22 more valuable. Further, the question is whether X22′s observed anti‐inflammatory and antioxidant effects are related to decreased lipid levels.

In conclusion, the findings of this study confirm the cardioprotective role of X22 against PA and HFD‐induced inflammation, oxidative stress, hypertrophy and fibrosis both *in vivo* and *in vitro*. Also, Figure S2 showed that X22 is highly effective to protect H9c2 cells from LPS‐induced injuries, including NF‐kB activation, oxidative stress, hypertrophy, collagen‐4 overexpression and PARP activation. These data together indicated that the compound X22 is indeed protective *via* its anti‐inflammatory actions in cardiomyocytes. Thus, one thing is clear, imidazopyridine derivatives, such as X22, could be promising therapeutic options for the treatment of obesity‐related cardiac complications. While these results do provide a deeper understanding of the role Nrf2 and NF‐κB play in hyperlipidemia‐induced cardiac injury and provide support for targeting the Nrf2 and NF‐κB pathways in the treatment of obesity‐related complications, more information is needed to clarify the mechanism behind X22′s cardioprotective effects. In addition, it is very interesting that X22 could reduce obesity in HFD‐fed mice. Our future plan on X22 also contains drug development and mechanistic investigation of X22 as an anti‐obesity or hypolipidemic candidate.

## Conflicts of interest

The authors confirm that there are no conflicts of interest.

## Author contribution

G.L., Y.Z. and K.P. participated in research design; Y.Q., P.Z., Z.X., X.C. and K.L. conducted experiments; Y.Q., Z.G. and X.C. contributed new reagents or analytic tools; G.L. and Y.Q. performed data analysis; P.Z. and X.L. wrote or contributed to the writing of the manuscript.

## Supporting information


**Figure S1** X22 treatment alone does not induce any changes of phenotype in H9c2 cells.
**Figure S2** X22 prevents lipopolysaccharide (LPS)‐induced inflammatory, oxidative stress, hypertrophy, fibrosis and apoptosis in H9c2 cells.
**Table S1** Primer sequences for real‐time quantitative PCR.
