# Supplementary material for: Inhibition of inflammation and oxidative stress by an imidazopyridine derivative X22 prevents heart injury from obesity
Source: J Cell Mol Med. 2016 Mar 28;20(8):1427–42. doi: 10.1111/jcmm.12832 (PMC4956940; doi:10.1111/jcmm.12832)
Supplement: Supplementary file 1 — Figure S1 X22 treatment alone does not induce any changes of phenotype in H9c2 cells. Figure S2 X22 prevents lipopolysaccharide (LPS)‐induced inflammatory, oxidative stress, hypertrophy, fibrosis and apoptosis in H9c2 cells. Table S1 Primer sequences for real‐time quantitative PCR. [file JCMM-20-1427-s001.doc]

**Inhibition of inflammation and oxidative stress by an imidazopyridine derivative X22 prevents heart injury from obesity**

Yuanyuan Qian^1,#^, Yali Zhang^1,#^, Peng Zhong^1,2^, Kesong Peng^1^, Zheng Xu^1^, Xuemei Chen^1^, Kongqin Lu^1^, Gaozhi Chen^1^, Xiaokun Li^1^, Guang Liang^1,*^

^1^Chemical Biology Research Center, School of Pharmaceutical Sciences, Wenzhou Medical University, Wenzhou, Zhejiang, China

^2^Department of Cardiology, the 5^th^ Affiliated Hospital of Wenzhou Medical University, Lishui, Zhejiang, China

**Running Title:** X22 attenuates obesity-induced heart injury

**#** These authors contribute equally to this work.

***Corresponding author:**

Guang Liang, Ph.D, at the Chemical Biology Research Center, School of Pharmaceutical Sciences, Wenzhou Medical University, Wenzhou 325035, China, Tel/Fax: +86-577-86699396; E-mail: [wzmcliangguang@163.com](mailto:wzmcliangguang@163.com)

**Table S1 Primer sequences for real-time quantitative PCR**

| Gene | species | Forward primer | Reverse primer |
| --- | --- | --- | --- |
| β-actin | Rat | AAGTCCCTCACCCTCCCAAAAG | AAGCAATGCTGTCACCTTCCC |
| TNF-α | Rat | TACTCCCAGGTTCTCTTCAAGG | GGAGGCTGACTTTCTCCTGGTA |
| IL-1β | Rat | CACCTCTCAAGCAGAGCACAG | GGGTTCCATGGTGAAGTCAAC |
| IL-6 | Rat | GAGTTGTGCAATGGCAATTC | ACTCCAGAAGACCAGAGCAG |
| ICAM-1 | Rat | AGATCATACGGGTTTGGGCTTC | TATGACTCGTGAAAGAAATCAGCTC |
| VCAM-1 | Rat | TTTGCAAGAAAAGCCAACATGAAAG | TCTCCAACAGTTCAGACGTTAGC |
| HO-1 | Rat | TCTATCGTGCTCGCATGAAC | CAGCTCCTCAAACAGCTCAA |
| NQO-1 | Rat | ACCTTGCTTTCCATCACCAC | CAAAGGCGAAAACTGAAAGC |
| GCLC | Rat | ATGCAGTATTCTGAACTACC | ACAAACTCAGATTCACCTAC |
| GCLM | Rat | AAGCCCAGGAGTGGGTGCCA | GCTTCCTGTGAGTGCGCTGCT |
| Nrf-2 | Rat | ACTGTCCCCAGCCCAGAGGC | CCAGGCGGTGGGTCTCCGTA |
| ANP | Rat | CTGCTAGACCACCTGGAGGA | AAGCTGTTGCAGCCTAGTCC |
| BNP | Rat | GATCCAGGAGAGACTTCGAAA | CGGTCTATCTTCTGCCCAA |
| TGF-β | Rat | GCAACAACGCAATCTATGAC | CCTGTATTCCGTCTCCTT |
| Collagen 1 | Rat | GACATCCCTGAAGTCAGCTGC | TCCCTTGGGTCCCTCGAC |

**Figure S1.** X22 treatment alone does not induce any changes of phenotype in H9c2 cells. An immunofluorescence assay was conducted to detect the expression and distribution of NF-κB p65 in H9c2 cells. Our results showed that X22 cannot activate NF-κB p65 nuclear translocation (A). Using DCFH-HA (for H_2_O_2_^-^) probes and SOD kit, we were able to show that ROS production was not increased (B and C). The cells were pretreated with X22 at 20 μM for 8 h. (D) X22 cannot induce hypertrophy in H9c2 cells. The effect of X22 on fibrosis was determined using protein expression of collagen IV, which revealed that X22 did not increase collagen IV (E). We further determined the protective effect of X22 on apoptosis in H9c2 cells. H9c2 cells were pretreated with X22 at 20 μM for 36 h. X22 did not induced apoptosis in H9c2 cells (F). For all experiments, n=3 separate determination.

*Supplementary method: SOD analysis.* The superoxide dismutase (SOD) levels in H9C2 cells were determined using commercially available kits according to the manufacturer's instructions (Beyotime Biotech, Nantong, China).

**Figure S2.** X22 prevents lipopolysaccharide (LPS)-induced inflammatory, oxidative stress, hypertrophy, fibrosis and apoptosis in H9c2 cells. An immunofluorescence assay was conducted to detect the expression and distribution of NF-κB p65 in H9c2 cells. In LPS-treated cells, NF-κB p65 accumulated in the nuclei, while X22 pretreatment reversed LPS-induced p65 nuclear translocation (A). Using DCFH-HA (for H_2_O_2_^-^) probes and GSH/GSSG kit, we found that ROS production was significantly increased following LPS treatment (0.5 mg/mL LPS for 8 h). However, pre-treatment with X22 at 20 μM for 1 h significantly decreased ROS production (B and C). The cells were pretreated with X22 at 20 μM for 1 h and then incubated with LPS at 0.5 mg/mL for 8 h. (D) X22 significantly suppressed LPS-induced hypertrophy in H9c2 cells (** p<0.01). The effect of X22 on fibrosis was determined using western blot analysis for protein expression of collagen IV, which revealed that X22 significantly reduced LPS-induced increase in collagen IV (E). We further determined the protective effect of X22 on LPS-induced apoptosis in H9c2 cells. H9c2 cells were pretreated with X22 at 20 μM for 1 h and then incubated with 0.5 mg/mL LPS for 36 h. LPS-induced apoptosis in H9c2 cells, X22 significantly reduced LPS-induced increase in cleave-PARP (F). For all experiments, n=3 separate determination.
